# Editorial: Biomarkers in head and neck cancer: genomic insights and nuclear medicine innovations

**DOI:** 10.3389/fmed.2026.1867004

**Published:** 2026-05-25

**Authors:** Sujith Baliga, Priyanka Bhateja

**Affiliations:** 1 Radiation Oncology, Winship Cancer Institute of Emory University, Atlanta, GA, United States; 2 Medical Oncology, James Cancer Center, Ohio State University, Columbus, OH, United States

**Keywords:** biomarkers, immunotherapy, inflammation, nomogram, nutritional index

## Introduction

The current issue focuses on biomarkers beyond clinical staging that may predict treatment outcomes in head and neck cancer. Locally advanced head and neck cancer has known predictive markers, including smoking and alcohol history, virally mediated pathology, and well-known genomic alterations such as p53 mutations. The immune inflammation score and nutritional index are widely recognized to reflect disease activity, and the neutrophil–lymphocyte (NL) ratio has been validated in several cancers as a predictor of response to immunotherapy agents (1).

## Biomarkers in head and neck cancer

PDL-1 expression as a biomarker of response to immunotherapy has been validated in recurrent/metastatic head and neck cancer (2). Xu et al. performed a meta-analysis aimed at identifying a PDL-1 threshold predictive of the efficacy of immune checkpoint inhibitors in recurrent/metastatic nasopharyngeal cancer. They pooled a total of 1,312 patients from 14 studies, two of which included immunotherapy in the frontline setting and 12 in later lines of treatment. Addition of immunotherapy led to an improvement in progression-free survival (PFS) in the frontline setting for patients with both PDL-1 scores >= 1 and PDL-1 scores < 1. In the subsequent-line setting, a PDL-1 score of ≥1 was associated with higher response rates, with an ORR of 0.37 vs. 0.22 in those with a PDL-1 score < 1 (*p* < 0.01).

Immune inflammation indexes could also reflect the tumor microenvironment and outcomes in the curative setting. Wang Y. et al. investigated a large single-center retrospective cohort of patients undergoing curative treatment and explored variables assessed at baseline, within 7 days before the initiation of surgery, radiation, or chemoradiation. They developed a systemic immune inflammation nutrition score using six biomarkers: The platelet–lymphocyte ratio (PLR), prognostic nutritional index (PNI), systemic immune inflammation index (SII), albumin–bilirubin index (ALBI), fibrinogen (FIB), and monocyte count. A majority of patients in this cohort had laryngeal cancer, followed by hypopharyngeal cancer, and two-thirds of the population had no nodal disease. There were no differences in clinicopathological parameters between the training and internal validation cohorts. Within the limitations of a retrospective cohort comprising early and locally advanced laryngeal and hypopharyngeal cancers treated with varying modalities with curative intent, the SIIN score showed good predictive value for 3-year OS (AUC 0.736) and 5-year OS (AUC 0.700). However, the SIIN score was based on a single time point, and variability or response to treatment was not evaluated.

In another study, Wang W.-Y. et al., performed a rigorous meta-analysis of perineural invasion involving 27 studies encompassing 4,000 patients and demonstrated that a low PNI was associated with an increased hazard for disease-free survival (DFS), progression-free survival (PFS), and cancer-specific survival. Importantly, these results were consistent across different treatment modalities, including chemoradiotherapy (CRT), surgery, and ICI-based immunotherapy. The authors posit that PNI may be related to lymphocytopenia, a compromised immune system, and an adverse tumor microenvironment. Future studies will need to explore whether these underlying mechanisms are supported by biological data.

Although Wang Y. et al. focused on a nutritional index, Ji et al. evaluated the role of the integrin alpha (ITGA) gene across 33 tumor types using The Cancer Genome Atlas. The investigators found that abnormal expression of ITGA genes was present in most tumor types, including head and neck squamous cell carcinoma (HNSCC), where increased expression correlated with poorer prognosis. Interestingly, within HNSCC, there was high expression of ITGA3, ITGA5, and ITGA6, which correlated with several clinical and pathological risk factors, including disease stage, lymph node metastasis, and poor survival in OS and DFS endpoints across 79 tissue specimens. These findings suggest that ITGA3, ITGA5, and ITGA6 may serve as prognostic biomarkers, supporting their potential use in clinical risk prediction.

These studies demonstrate how HNSCC is influenced not only by the inherent molecular features of the tumor but also by the immunogenicity of the patient. These prognostic biomarkers may allow clinicians to consider treatment intensification in patients with poor prognostic features.

## Future perspectives

Biomarkers and nomograms continue to be explored for outcome prediction (3). Nomograms such as the one developed by Wang W.-Y. et al. combine multiple parameters derived from readily available laboratory values and help overcome some of the limitations associated with tissue-based immune or genomic markers. One major limitation is the reproducibility and external validation of these nomograms, especially in prospective studies. Research topics such as those highlighted in this issue can aid in integrating varied biomarkers, ranging from genomic and immune markers to laboratory-based tests, and may guide innovation and improve the characterization of subgroups. With the advancement of treatments for HNSCC, biomarkers remain important but still elusive for treatment selection and individualization. An ideal biomarker would be both prognostic and predictive of treatment outcomes, in addition to having the ability to categorize patients into well-defined, distinct subgroups. This will likely be achieved through a multi-omics approach, coupled with radiomics and accelerated by machine learning (4).

As outlined in the meta-analysis by Xu et al., studies showing a lack of predictive value for certain biomarkers are as important as those identifying predictive biomarkers, especially in highly inflamed tumors such as nasopharyngeal cancer, where patients should not be excluded from effective treatment options. Lack of PDL-1 expression should not be used as an exclusion criterion in recurrent/metastatic nasopharyngeal cancer for first-line treatment with immune checkpoint inhibitors (5).

## Conclusion

The present Research Topic connects nutritional indexes and PDL-1 scores with the prediction of treatment outcomes. These contributions highlight the role of biomarkers and the need for a multipronged approach to aid in treatment selection.
